# The increased risk for thromboembolism pre-cystectomy in patients undergoing neoadjuvant chemotherapy for muscle-invasive urinary bladder cancer is mainly due to central venous access: a multicenter evaluation

**DOI:** 10.1007/s11255-019-02338-4

**Published:** 2019-11-19

**Authors:** Kristoffer Ottosson, Sofia Pelander, Markus Johansson, Ylva Huge, Firas Aljabery, Amir Sherif

**Affiliations:** 1 Department of Surgical and Perioperative Sciences, Urology and Andrology, Umeå University, 901 85 Umeå, Sweden; 2 Division of Urology, Department of Clinical and Experimental Medicine, Linköping University, Linköping, Sweden; 3 Department of Urology, Sundsvall Hospital, Sundsvall, Sweden

**Keywords:** Complications, Cystectomy, Neoadjuvant therapy, Thromboembolism, Urinary bladder neoplasms

## Abstract

**Purpose:**

To investigate if patients receiving neoadjuvant chemotherapy (NAC) for muscle-invasive bladder cancer (MIBC) had an increased risk of thromboembolic events (TEE) and to evaluate when these events occur on a timeline starting from 6 months pre-cystectomy, during NAC-administration and 60 months post-cystectomy.

**Methods:**

Two hundred and fifty five patients undergoing radical cystectomy during 2009–2014 at three Swedish cystectomy centers (Umeå, Linköping and Sundsvall) were in-detail reviewed retrospectively, using individual medical records. One hundred and twenty nine patients were ineligible for analysis. NAC patients (*n* = 67) were compared to NAC-naïve NAC-eligible patients (*n* = 59). The occurrence of TEE was divided into different periods pre-cystectomy and post-cystectomy. Statistical analyses included Chi-squared and logistical regression tests.

**Results:**

Significant associations were found between receiving NAC and acquiring a TEE during NAC therapy pre-cystectomy. All but one pre-cystectomy event was venous and all but one of the patients received NAC. 31% (14/45) of TEEs occurred pre-cystectomy. The incidence of TEEs pre-cystectomy in NAC-naive NAC-eligible patients was only 10% (2/20), whereas the incidence of TEEs in NAC patients occurred pre-cystectomy in 48% (12/25) and 11/12 incidents were detected during NAC therapy—this including 7/11 (64%) incidents affecting veins in anatomical conjunction with the placement of central venous access for chemotherapy administration.

**Conclusions:**

There is a significantly increased risk for TEE pre-cystectomy during chemotherapy administration in MIBC patients receiving NAC, compared to the risk in NAC-naïve NAC-eligible MIBC patients. In 64% of the pre-RC TEEs in NAC patients, there was a clinical connection to placement of central venous access.

## Introduction

Urinary bladder cancer (UBC) is the fourth most common malignancy in men and eighth most common in women, in the western world [1]. While most newly diagnosed patients have non-muscle-invasive bladder cancer, urothelial muscle-invasive bladder cancer (MIBC) accounts for approximately 25% of new cases, with a 5-year overall survival (OS) of approximately 50% in stages cT2-T4 after radical cystectomy [2]. Cisplatin-based neoadjuvant combination chemotherapy (NAC) for MIBC is a treatment, first introduced internationally and nationally (Sweden) in the mid-2000s with the intention to eradicate micrometastatic disease at the best point of time. The introduction of NAC had been preceded by reliable randomized prospective studies showing that the treatment had conveyed survival benefits equivalent to a 5–8% absolute improvement in 5 years median time, compared to local treatment (RC) only [3, 4]. NAC has been shown to significantly increase OS for a chemosensitive subgroup of these patients, with an absolute risk reduction (ARR) of 31% in completely downstaged patients (pT0N0M0) at 5 years median observation time [5]. Standard treatment is three (and in some centers four) cycles of NAC followed by radical cystectomy with curative intent (RC).

Data from radical cystectomy studies have shown a wide variation of venous thromboembolism (VTE) with incidences, ranging from 2.9 to 24.4% [6, 7]. The wide range of events is likely due to the different ways of classifying VTE. For instance, in one study [7], all patients underwent ultrasound screening of the lower extremities to detect VTE, which is not in line with clinical practice. Among 16 VTEs found, only one was clinically symptomatic, leading to difficulties in comparing this study with those classifying TEEs as clinically symptomatic or incidental such as in the study of Duivenvoorden et al. [8]. In addition, a large Danish study of 13,809 patients showed that risks for VTE are particularly high for bladder cancer patients, especially post-RC with a 70-fold increase in frequency [9]. Furthermore, VTE in connection with RC has been associated with an increase in mortality and worse long-time survival [10, 11]. Cancer itself is considered being a risk factor likely due to immobilization [12, 13] and the hypercoagulable state induced by malignancy [14]. Patients undergoing neoadjuvant NAC and RC have also been shown to have an increased risk for thromboembolic events (TEEs) [8, 15]. The study of Zareba et al. evaluated 202 patients and found the risk ratio of 3.39 for TEEs in patients treated with NAC and RC compared to RC only [15]. Duivenvoorden et al. described 761 MIBC patients undergoing NAC and detected an overall incidence of TEE in 14%, of which 58% developed this complication preoperatively [8]. Yet, that study did not specify the NAC-naïve patient cohort in terms of NAC eligibility or even cTNM for both investigated retrospective cohorts. The multivariable analysis did not contain comorbidity and further, the preoperative period was not clearly defined. Even if NAC is recommended in European guidelines [16], as well as in Swedish guidelines for patients with urothelial bladder cancer staged T2-T4aNM0 [17] until now, there are no recommendations pertaining to thromboprophylaxis in MIBC patients undergoing NAC.

Our aims were to evaluate if NAC patients had an increased risk for TEEs compared to a well-matched control group and to investigate what the distribution of TEEs was over time from 6 months before the final TUR-B until 5 years post-cystectomy.

## Methods

### Patient population

**Fig. 1:**
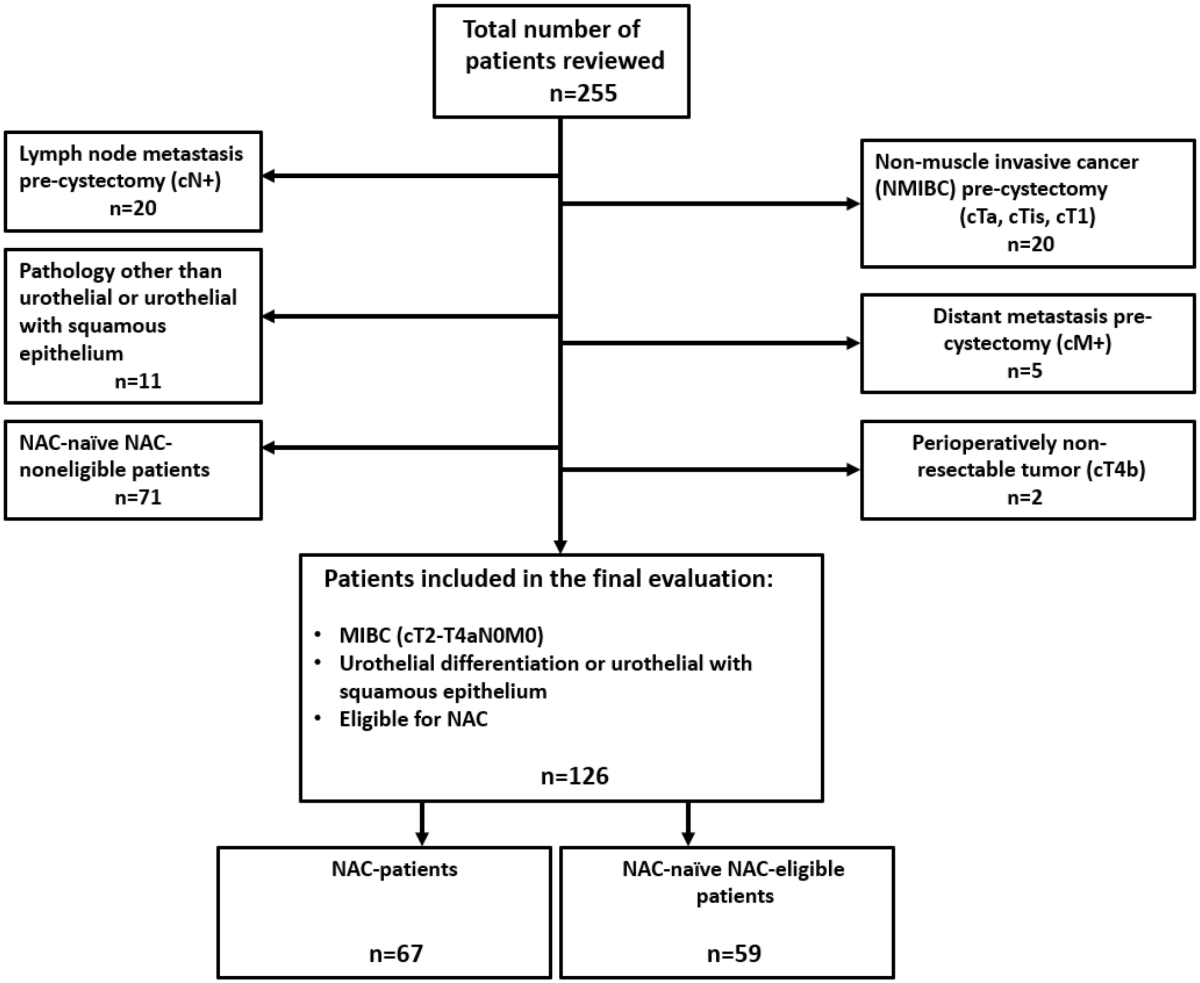
Flowchart visualizing the inclusion and exclusion processes, resulting in 126 analyzed patients

The primary patient population in this study constituted of all patients that underwent RC at the university hospitals of Umeå and Linköping, as well as at the County hospital of Sundsvall between the years 2009–2014 (*n* = 255). Exclusion criteria for qualifying to final analysis are outlined in the flowchart (Fig. 1). Finally, there were two well-matched groups, NAC patients and NAC-naïve NAC-eligible patients; with totally 126 patients to analyze (baseline patient characteristics seen in Tables 1, 2). Most of the NAC patients (76%) received MVAC-HD or MVAC and 76.5% received three or four preplanned cycles of treatment (Table 3).

**Table 1:** Basic descriptive data of patient characteristics with continuous variables for the patient population

	NAC-eligible patients (*n* = 59)				NAC patients (*n* = 67)				Total (*n* = 126)			
	Min	Max	Mean	Median	Min	Max	Mean	Median	Min	Max	Mean	Median
Age	42	75	65	67	44	77	67	68	42	77	66	68
CACI	2	11	5	5	2	9	5	5	2	11	5	5
Number of dissected lymph nodes	0	83	25	20	0	27	12	12	0	83	18	15
No. of metastastatic lymph nodes found postoperatively	0	11	1	0	0	3	0	0	0	11	1	0
Admission time for RC (days)	7	56	17	16	4	59	17	15	4	59	17	15
Hb pre-RC within 48 h	109	164	142	145	90	167	117	116	90	167	126	123
Total operation time (min)	282	796	457	445	203	585	389	373	203	796	424	405
Perioperative bleeding (ml)	50	8000	1959	1300	200	2800	993	950	50	8000	1449	1000
Erythrocyte units during admission for RC	0	17	4	2	0	19	4	3	0	19	4	3
Total number of TEEs	0	2	0	0	0	3	0	0	0	3	0	0

**Table 2:** Basic patient characteristics over NAC-naïve NAC-eligible and NAC patients with urothelial MIBC

	NAC-eligible patients (*n* = 59)	NAC patients (*n* = 67)	Total (*n* = 126)
Gender			
Female	15	13	28
Male	44	54	98
Year of cystectomy			
2009	15	3	18
2010	18	10	28
2011	13	10	23
2012	7	10	17
2013	5	14	19
2014	1	20	21
Cystectomy center			
Linköping	32	4	36
Sundsvall	10	11	21
Umeå	17	52	69
cT-stage			
T2	37	30	67
T3	21	31	52
T4a	1	6	7
pT-stage			
T0	7	22	29
Ta, Tis, T1	1	10	11
T2	14	18	32
T3	27	7	34
T4a	8	5	13
T4b	2	3	5
pN-stage			
N0	41	56	97
N1	5	4	9
N2	12	6	18
N3	1	0	1
pM-stage			
M0	58	67	125
M1	1	0	1
Concomitant prostate cancer			
No	32	37	69
Yes	12	17	29

**Table 3:** Most of the NAC patients; 51/67 (76%) received MVAC-HD or MVAC and 76.5% received three or four preplanned cycles of treatment

Variable	Total *n *= 67
NAC treatment	
Cisplatin-Gemzar	3 (4.5)
MVAC-HD/MVAC	51 (76)
MVEC-HD/MVEC	11 (16.5)
Carboplatin-Gemzar	2 (3)
Number of NAC cycles	
One cycle	3 (4.5)
Two cycles	13 (19)
Three cycles	40 (60)
Four cycles	11 (16.5)

Data are shown as *n* (%)

### NAC eligibility

An important part of this study was to generate a matching control group for comparison with the NAC patients, enabling us to assess the risk of getting a TEE without too many confounders. This was performed by evaluating and dividing the patients who were NAC-naïve (*n* = 188) into two different groups: NAC-eligible (*n* = 59) and NAC-noneligible (*n* = 129) and only the former group was considered as the control group. Since NAC is a relatively novel form of treatment for urothelial MIBC patients, many NAC-eligible patients, especially in the first years of the series were non-receivers of NAC. Not because they were not a good fit for chemo, but because the treatment was just not widespread in common practice yet. An example of that is from the largest center in this series (NUS/Umeå), in which NAC for MIBC in 2009 was utilized in less than 40% of eligible patients followed by a steady increase to 81.3% in 2014 [18]. Hence the patients of the NAC-naïve NAC-eligible groups were defined as patients who should have received NAC according to current guidelines.

Definition of the patients’ eligibility for NAC, in the study, was performed for each patient together with a senior urologist and collaborator in the research group at Umeå University. We determined eligibility, kidney function, age, and CACI (Charlson age comorbidity index) [19]. Patients scoring outside the limits set for either category were considered NAC-noneligible, and therefore excluded from the analyses. As a rule of thumb, patients had to be 75 years or younger have a GFR of > 50 and a CACI of 6 or less to be considered eligible. In addition; if there was formal advice from a Multidisciplinary Team (MDT) conference in individual patients, actual conference recommendations were always used for defining individual eligibility for NAC.

### Data collection, our definitions of TEE and observation time

**Fig. 2:**
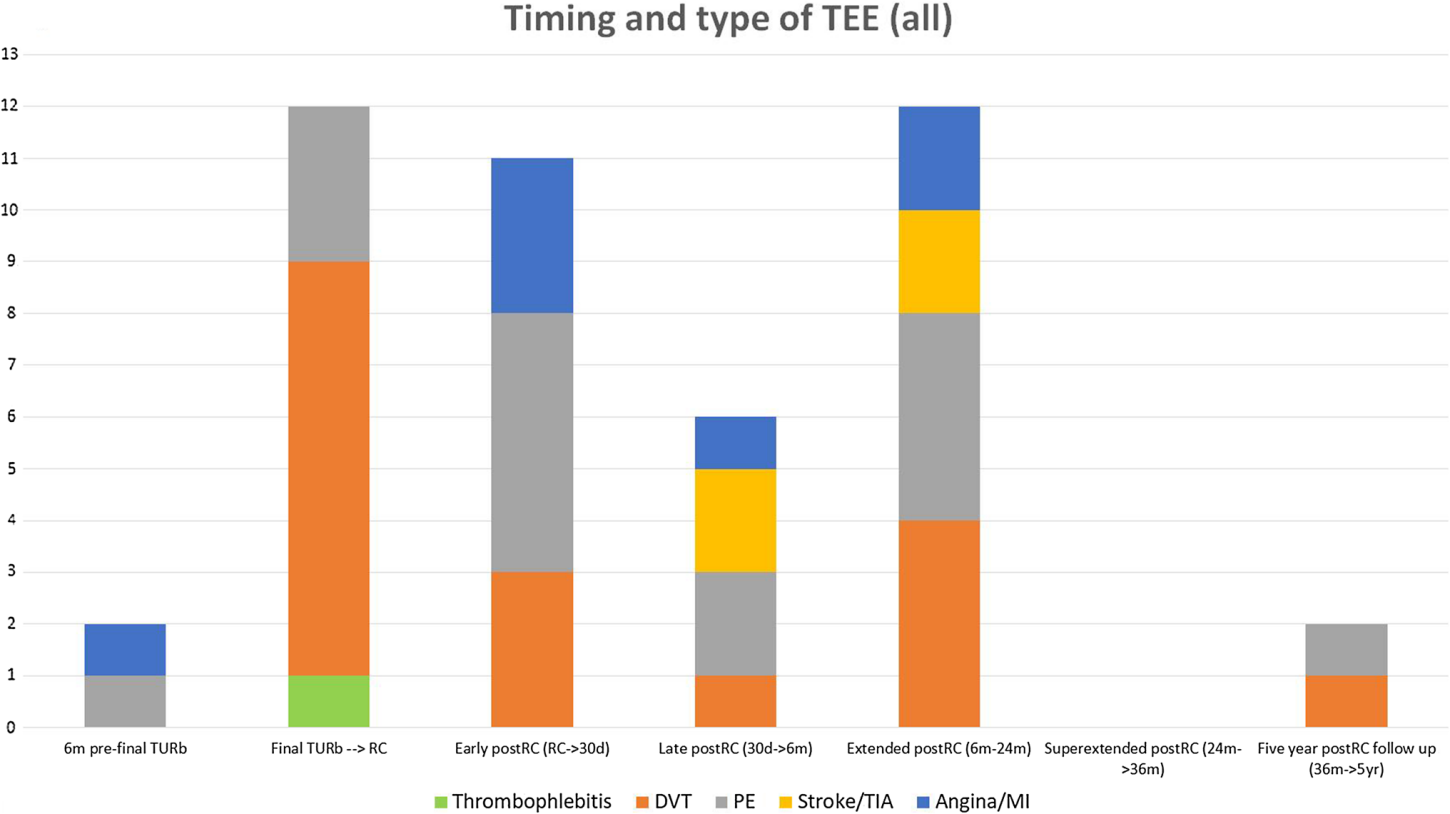
Types of TEEs and their exact timing over the full observation time for all patients (*n *= 126). Thrity four patients had at least one TEE giving a total amount of 45 TEEs (considering that some patients had more than one TEE)

Data were collected from individual medical records (Tables 1, 2). TEEs were defined as any type of thromboembolic event, both venous and arterial, such as myocardial infarction (MI), ischemic stroke/TIA, deep venous thrombosis (DVT), pulmonary embolism (PE), or thrombophlebitis. The observation time started 6 months before the final TUR-B and ended either 5 years post-cystectomy or at death (Fig. 2). The cutoff date for all observations of TEEs, as well as death was 31st December 2016. Patients who got a TEE at any point during the observation time were registered as single cases and in addition, the total number of TEEs was noted separately, accounting for some patients having more than one TEE. Within the observation time, TEEs were also registered in smaller periods; 6 months before the final TUR-B, between the final TUR-B and RC (thus including the period when the NAC patients received chemotherapy), early postoperative, late postoperative, extended postoperative, super extended postoperative, and 5-year follow up. The exact timing of a TEE was registered, making it possible to see its distribution over time. Additionally, TEEs were registered cumulatively—if a patient had a TEE within 24 months postoperatively, he or she was considered having had a TEE within 36 months and 5 years postoperatively, as well. The 5-year follow up category was therefore used for patients that had a TEE at any time during a 5-year postoperative period.

### Statistical analyses

All statistical analyses were conducted using IBM SPSS statistics version 25. A Chi-squared test was used for categorical variables for a swift overview of which variables, and especially, which follow-up times had a possible association with TEE incidence. Further, due to the relatively small number of patients, the possibility to adjust for certain variables was limited. Hence, if a significant result was found using Chi-squared test (*p* < 0.05), test for logistic regression was carried out and if possible, certain variables were adjusted for. The comparisons were between NAC patients and NAC-naïve NAC-eligible patients to investigate if NAC patients were at an increased risk of getting TEEs.

For continuous variables test, logistic regression was conducted and did not consider if the patient had received NAC or not, this to evaluate if there were any other subgroups that were at an increased risk of getting TEEs.

### Ethical approval

The study was approved by the regional ethics board in Umeå: EPN-Umeå, dnr; 2013/463-31 M and amendments 2016/129-32 M and 2016/403-32. The study conforms to the provisions of the Declaration of Helsinki (as revised in Fortaleza, Brazil, October 2013). The regional ethics board had specifically decided that informed consent from the participants was to be considered redundant, especially due to the high mortality in MIBC, as well as due to the retrospective nature of the study.

## Results

### Description of observed TEEs

**Fig. 3:**
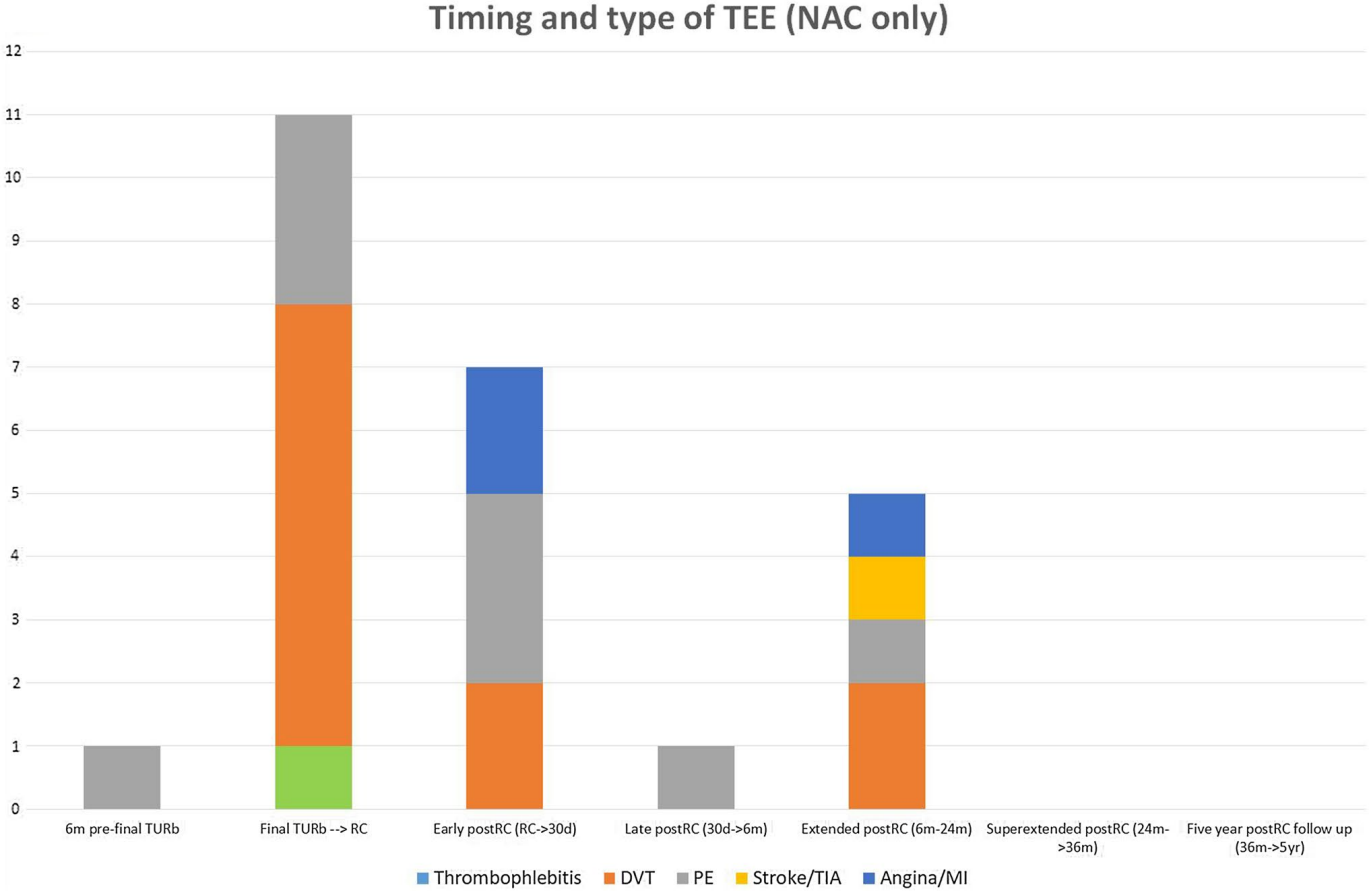
Types of TEEs and their exact timing over the full observation time for NAC patients (*n* = 67); 17 NAC patients had a TEE and the total amount of TEEs was 25 (considering that some patients had more than one TEE)

**Fig. 4:**
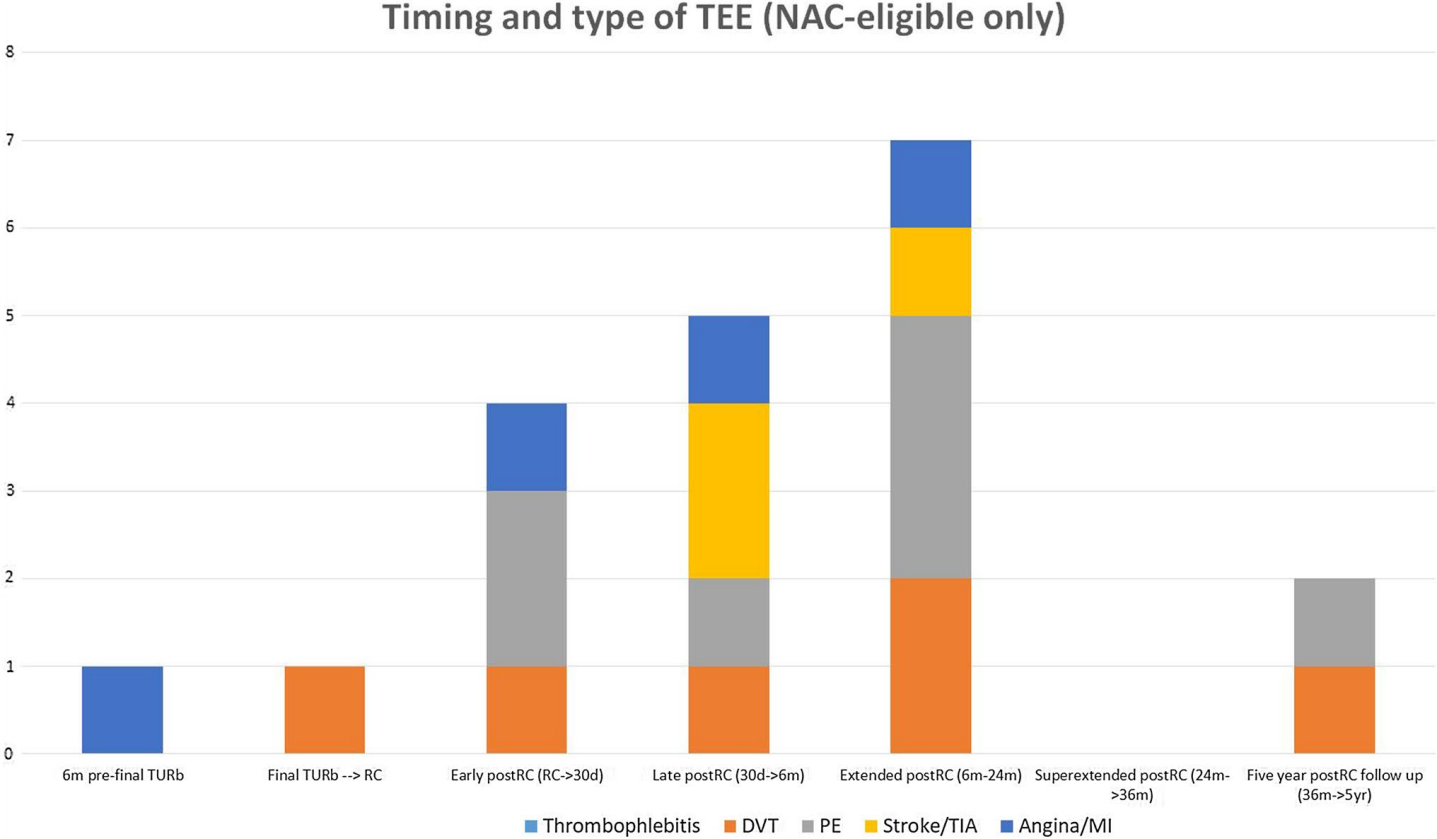
Types of TEEs and their exact timing over the full observation time for NAC-eligible patients (*n* = 59); 17 patients had at least one TEE and the total amount of TEEs was 20 (considering that some patients had more than one TEE)

In total, 34 patients had a TEE at any point during the observation time. Seventeen of them were NAC patients and 17 were NAC-naïve NAC-eligible patients. Totally 45 TEEs were detected, considering that eight patients had more than one TEE, giving an overall TEE incidence of 35%. Through the complete observation time, the incidence of TEEs in NAC-patients was 37.3% and in NAC-naïve NAC-eligible patients it was 33.9%. The period between the final TUR-B → cystectomy and further the period between 6 and 24 months postoperatively contained the highest number of TEEs for NAC patients. Among all the TEEs that occurred for NAC patients (in total 25 events), 48% occured in the final TUR-B → cystectomy period plus in the pre-TUR-B period (12/25), whereas only 10% of all the TEEs that occurred for NAC-naïve NAC-eligible patients (2/20 events), were identified in the same two pre-cystectomy periods. All 11 events in the NAC cohort were venous in the final TUR-B → cystectomy period. If a patient had a TEE in direct connection with the establishment of a central venous access (CVC, PICC-line, port-a-cath), this was counted as a DVT (deep venous thrombosis). DVTs were detected in 6/11 events in the NAC cohort and a thrombophlebitis in 1/11 during the TUR-B → cystectomy period. One of eight NAC patients had both a DVT and a PE and a total of three patients had four incidents of PE in that period (Table 4). NAC-naïve NAC-eligible patients had more postoperative TEEs (18) compared to NAC patients (13). Post-cystectomy, arterial TEEs also occurred in addition to venous, where MI was the most frequent, with totally six events, three in each cohort (Figs. 2, 3, 4).

**Table 4:** Description over the eight NAC patients who experienced a total of 11 TEEs, during resp. chemotherapy periods

	Deep venous thrombosis	Thrombophlebitis	Pulmonary embolism
Patient A			1
Patient B	1		
Patient C	1		1
Patient D		1	
Patient E	1		
Patient F	1		
Patient G	1		
Patient H	1		2
Total	6	1	4

Two patients had during the course of time both DVT and PE and one of the patients (patient H) had repeat PE later on during the chemotherapy period. In all six incidents of DVT, the origin was the deep venous system in anatomical conjunction with resp. CVA. In the patient with thrombophlebitis, it also appeared on the same side as the CVA and in anatomical proximity. All eight patients received antithrombotic treatment following a diagnosis of TEE. Three of eight patients discontinued planned NAC and instead proceeded to RC. The remaining five patients received all NAC cycles as initially was planned before they underwent RC

### TEE incidence analyzed with categorical variables: NAC patients versus NAC-naïve NAC-eligible patients

The TEE incidence was compared between the two groups assessing both the different periods (between the final TUR-B → cystectomy and from the cystectomy to 30 days, 6 months, 24 months, 36 months, and 5 years postoperatively, respectively), as well as for the total observation time. For all conducted statistical analyses, see Tables 5, 6.

**Table 5:** Tests conducted for categorical variables

	NAC		TEE 6 months pre-final TUR-B	
	Fisher’s exact Test		Fisher’s exact Test	
	Exact Significance (2-s)	Exact Significance (1-s)	Exact Significance (2-s)	Exact Significance (1-s)
TEEs at different time periods				
6-month pre-final TUR-B	X	X	X	X
Final TUR-B → RC	***0.019***	***0.014***	1.000	0.847
RC → 30 days	0.489	0.275	1.000	0.826
RC → 6 months	0.056	***0.031***	1.000	0.894
RC → 24 months	0.094	0.064	1.000	0.705
RC → 36 months	0.232	0.133	1.000	0.736
RC → 5 years	0.434	0.247	1.000	0.645
Final TUR-B → 5 years	0.407	0.205	0.433	0.433
Full observation time	X	X	X	X

For the period between the final TUR-B → cystectomy, an association could be found between receiving NAC and the risk of getting a TEE (*p *= 0.019, Fisher’s Exact Test), (*p* = 0.015, Pearson Chi-Square - data not shown)

**Table 6:** Logistical regression tests conducted, testing NAC against TEEs for the period between the final TUR-B → cystectomy

	Variables tested against TEEs		
	NAC		
	*p* value	OR	95% CI
Final TUR-B → RC	*0.040*	**9.000**	1.105–73.334
Final TUR-B → RC (adjusted for CACI)	*0.048*	**8.342**	1.019–68.271

The results remained significant, both unadjusted (OR = 9.000, 95% CI 1.105–73.334, *p *= 0.040), as well as adjusted for CACI (OR = 8.342, 95% CI 1.019–68.271, *p *= 0.048)

Two significant results were found. For the period between the final TUR-B → cystectomy, an association could be found between receiving NAC and the risk of getting a TEE (*p* = 0.015, Pearson Chi-squared and *p* = 0.019, Fisher’s exact test) (Table 5). Further, when conducting logistic regression for this period, results remained significant, both unadjusted (OR = 9.000, 95% CI 1.105–73.334, *p* = 0.040), as well as adjusted for CACI (OR = 8.342, 95% CI 1.019–68.271, *p* = 0.048) (Table 6).

For the 6-month postoperative category (RC → 6 months post-RC), an association between NAC and TEE could be found using Pearson Chi-squared test (*p* = 0.024), though not with Fisher’s exact test (*p* = 0.056) (Table 5). When carrying out the test for logistic regression, significance was also lost (*p* = 0.997) (data not shown). For all other periods, no significant associations could be shown between NAC and TEE incidence.

### TEE incidence with other categorical variables

For the 6-month before the final TUR-B period, only two incidents of TEEs were identified and no significant association could be shown when compared with later periods (Table 5).

### TEE incidence with continuous variables

Perioperative bleeding and total operation time were tested against TEE incidence, both in the total postoperative time and for the different periods. No significant results were found (data not shown).

## Discussion

Neoadjuvant cisplatin-based combination chemotherapy with radical cystectomy is the treatment of choice in medically fit patients with urothelial MIBC [16] and confers a significant impact on improved long-time survival, especially in patients with complete responses (pT0N0M0) [5]. Yet, it is warranted to identify significant side effects, as well as complications to this otherwise successful treatment. Naturally, improvement and optimization of different treatment aspects would hopefully prove beneficial for the patients. Other retrospective studies have suggested that NAC is associated with an increased risk for TEEs [8, 15] and we aimed to investigate the matters comparing NAC patients with meticulously well-matched NAC-naïve, but yet NAC-eligible patients, in a wider frame of time, stretching from 6 months pre-final TUR-B to 5 years post-RC. By detailed evaluation of the individual medical records, we also aimed at trying to identify key factors that could be of importance.

In the study, we found that the overall TEE incidence for all MIBC patients (35%) was higher than, for example, in the investigations of Clement et al.; 2.9% or Dyer et al.; 24.4%) [6, 7]. Yet, the mentioned studies only evaluated VTE and not an expanded set of TEE definitions.

Duivenvoorden et al. found the overall incidence of TEEs for patients undergoing NAC and RC to be 14% [8], where 58% occurred preoperatively compared to this study’s higher overall incidence of 35% and lower preoperative incidence of 31%, respectively. It is possible that these results would be more in line with each other, if our study had a larger sample of patients. Duivenvoorden et al. also excluded patients already on anticoagulation drugs (*n* = 21) and used a shorter observation time (up to 6 months postoperatively), which are other examples of differences compared to this study and are possible explanations for the variations in incidence figures as well.

When patients in our study received their NAC treatment, all of them had been supplied with either kind of central venous access (CVA) for drug administration. NAC-naïve NAC-eligible patients naturally did not receive a CVA for preoperative chemotherapy. CVA placement would be a plausible explanation for the significant findings of higher TEE odds for NAC patients compared to NAC-naïve NAC-eligible patients during this period. A central venous access, when placed, causes local injury in the vein and is a common cause of DVT in the upper extremity [20]. A recently published retrospective study on 539 cancer patients who underwent chemotherapy (adjuvant and palliative, but not neoadjuvant) following a CVA placement showed that 7.2% developed VTE during the 1 year of follow up. The most interesting finding was that the patients were less likely to develop VTE, if they had been on an antiplatelet agent (OR 0.28, *p *= 0.03). Further, none of the patients on anticoagulation therapy had developed VTE [21].

Hereby we suggest that the significant results found in the period between the final TUR-B → cystectomy (i.e., mainly during the neoadjuvant chemotherapy period) could be explained by the presence of a CVA in NAC patients compared to in NAC-naïve NAC-eligible patients, as well as possibly the medication itself as an additional reason. Cancer itself is a risk factor likely due to immobilization [12, 13] and the hypercoagulable state induced by malignancy has been suggested as another risk factor [14]. Our findings could be used as hypothesis-generating for future retrospective trials on a larger scale, as well as for prospective randomized trials with focus on active TEE prophylaxis. Yet, the 95% confidence intervals unadjusted (1.105–73.334), as well as adjusted for CACI (1.019–68.271) for the results were rather large. This makes the findings somewhat unstable. Significant association between NAC and TEE was also found for the 6-month postoperative category when using Pearson Chi-squared test (data not shown), but with the other tests, however, significance was lost, thus making the finding unreliable. The fact that Fisher’s exact test, which was insignificant, is better suited for smaller data amounts such as this weakens that finding even further. General weaknesses with this study include its retrospective nature, as well as a rather small number of patients. Small changes or errors in the data could alter the results in a significant way. In short, we find that there is a significantly increased risk for TEE pre-cystectomy during the period of chemotherapy administration compared to the risks in NAC-naïve NAC-eligible MIBC patients. Plausible reasons are; placing of the central venous access, chemotherapy treatment, the malignancy, and combinations thereof. Randomized prospective trials with a focus on early TEE prophylaxis in the experimental arm would be of value.
